# A Retrospective, Observational and Descriptive Study of 111 Ventral Hernia Repairs: Is the Open Approach Already over the Hill?

**DOI:** 10.3390/jcm14020560

**Published:** 2025-01-16

**Authors:** Giorgio Ammerata, Giuseppe Currò, Giuseppe Sena, Michele Ammendola, Francesco Abbonante

**Affiliations:** 1 Science Health Department, General Surgery Unit, University “Magna Graecia” Medical School, 88100 Catanzaro, Italy; 2 Science Health Department, Digestive Surgery Unit, University “Magna Graecia” Medical School, 88100 Catanzaro, Italy; 3 Surgical Science Department, Plastic and Reconstructive Surgery Unit, “Pugliese-Ciaccio” Hospital, 88100 Catanzaro, Italy; franco.abbonante@gmail.com

**Keywords:** open ventral hernia repair (OVHR), Trabucco technique, long-term follow-up, chronic pain, recurrence rate

## Abstract

**Objectives:** Incisional ventral hernia repair remains a challenging surgery for abdominal wall surgeons. We report the results at 48 months post-surgery regarding open ventral hernia repair (OVHR), analyzing the recurrence rate and incidence of chronic pain. **Methods:** This was a retrospective, observational study of 111 consecutive patients who underwent OVHR. Between January 2017 and December 2019, patient data were collected from a database and classified by hernia type. Through questionnaires and clinical examinations, the recurrence rate and incidence of chronic pain (measured using the VAS score and a Likert scale) were obtained. **Results:** In all patients, the hernia repair was performed via an open approach. Long-term follow-up (48 months after surgery) revealed that 20% of patients experienced mild chronic pain alongside the flanks, and the recurrence rate was 5%. Moreover, long-term follow-up revealed the following secondary outcomes: movement limitations in sports were reported in 7% of patients, and movement limitations during long walking were reported in 11% of patients. **Conclusions:** Our technique for OVHR is a safe procedure with a low rate of recurrence and chronic pain. Our future aim is to organize a prospective study.

## 1. Introduction

Incisional hernia (I.H.) is a common complication after abdominal surgery. Its development depends on patient risk factors such as smoking, obesity, and collagen-related diseases [1,2].

Nowadays, the choice of surgical treatment for I.H. is varied, ranging from open to robotic procedures. Furthermore, the concept of abdominal wall repair has been revolutionized by mesh introduction.

Indeed, before mesh introduction, ventral hernias were repaired with direct suture techniques. For instance, a simple fascial closure, modified by Mayo with the overlap of fascial edges, the use of internal retention sutures, and the Maingot ‘Keel’ procedure represented the principal models. Moreover, the ‘Nuttall’ procedure involved the transposition of the rectus muscles and the enveloping fascia or the use of layered steel wires.

Nevertheless, the direct suture of defects led to wound-related problems such as infection, hematoma, stitches, and sinus and flap necrosis [3].

With the introduction of the knitted monofilament polypropylene mesh (Marlex) by Usher in 1963 [4] and polytetrafluoroethylene (PTFE) in 1972, the direct suture was gradually abandoned; on the other hand, there was the increasing development of different surgical options based on the position of the mesh. Nowadays, the choice of the mesh position varies; there are four possible sites, namely onlay, inlay, retromuscular or sublay, and intraperitoneal (IPOM), and all of these can be applied in an open, laparoscopic, or robotic approach.

### 1.1. Open Procedures

Regarding the onlay option, Chevrel first described this technique. The anterior layer of the rectus sheath was exposed at both sides through the resection of the skin and subcutaneous tissue. The anterior rectus sheaths were vertically incised 2 cm from their medial borders, creating two medial strips that were freed on their undersurfaces from the recti, mainly at the fibrous intersections that crossed the recti at three or four levels. The hernial sac was dissected and resected. The peritoneum was closed with a continuous absorbable suture. The medial edges of the defect were approximated with 2-0 non-absorbable sutures. The new linea alba was restored with two rows of interrupted non-absorbable U-sutures. This plasty was then reinforced by a prosthesis that was anchored 3–4 cm lateral to the medial border of the remaining rectus sheath [5].

Regarding the inlay procedure, its use is obsolete due to its position. In fact, this position could cause adhesion to the bowels; moreover, the inlay method does not restore the abdominal wall anatomy, creating significant tension between the mesh and fascial plane.

Another example of abdominal wall reconstruction is represented by the retromuscular or sublay technique, described by Rives and Stoppa. This technique is described in detail in Section 2.2.

In 1990, Ramirez reinforced the method of hernia repairing, introducing the concept of anterior component separation (ACS). This procedure allows the rectus, internal oblique, and transverse abdominis muscles to move medially through the division of the external oblique aponeurosis, elevating the rectus abdominis muscle from its posterior rectus sheath and then mobilizing the myofascial flap [6].

Currently, the preferred option for I.H. repair is characterized by the use of minimally invasive methods (laparoscopy and robotic) due to reduced postoperative pain, shorter hospital stays, and faster recovery for daily activities [7,8].

### 1.2. Laparoscopic Procedures

William and Leblack reported the first laparoscopic surgery for ventral incisional hernia in 1993 [9].

Over the years, the laparoscopic procedure has undergone different modifications. However, the use of laparoscopy presents several indications as follows: symptoms such as pain, abdominal enlargement, and the risk of incarceration, especially for hernias with a small neck that contains the bowel. Other indications are simultaneous surgeries (for example, cholecystectomy) and the re-recurrence of hernia.

On the other hand, contraindications include acute or emergency procedures (bowel obstruction), skin infections, ascites with Child class ‘C’ cirrhosis, those with loss of domain (because the contents of the hernial sac cannot be reduced), and open wounds (insufflation is impossible), where additional gastrointestinal surgery is required. There are several sites involved in I.H., including midline, lateral, and iliac incisions. Upper midline incisional hernias have a higher incidence compared to others. The type could depend on the configuration of the collagen bundles of the abdominal wall, which are oriented transversely, so a transverse suture line is mechanically more stable.

The laparoscopic technique requires the placement of serial trocars to repair the defect, eliminating adhesiolysis and reducing the contents of the hernial sac. Moreover, after the visualization of the hernial defect, the mesh is placed in the intraperitoneal (IPOM) space, overlapping the defect in all directions.

### 1.3. Robotic Procedures

Despite the widespread use of the laparoscopic technique, the robotic approach allows ergonomic movements through wristed instrumentation, which shows some advantages in minimally invasive hernia repair, such as the fixation of the mesh without the use of tacks and a strong technical ability to close the defect, which is typically a challenge even for laparoscopic surgeons [10,11,12].

Furthermore, several studies have been conducted regarding the safety and reproducibility of robotic systems. For example, Gonzalez et al. described the short-term outcomes after robotic repair in 368 ventral hernias, which were treated by five surgeons from four different institutions. The short-term outcomes (30 days after surgery) were represented by urinary retention, paralytic ileus, wound and mesh infections, and seroma requiring intervention. Despite the fact that seroma was the most common complication after surgery, this study highlighted how robotic procedures could be performed by inexpert surgeons with low complication rates [13].

With the advent of robotics, more complex hernias are being approached in a minimally invasive fashion. Some benefits are listed as follows: better fascial closure, the retrorectus placement of the mesh, rectus muscle release, and the intraperitoneal suturing of the mesh. The advantage of robotic hernia repair is the possibility to obtain the same quality as in traditional hernia repair, eliminating its perioperative morbidity. Although fascia closure and the position of the mesh are debatable, robotic-assisted surgery has offered surgeons more options to treat abdominal wall diseases. For instance, IPOM and IPOM-plus (IPOM with intracorporeal fascial closure) have typically been the two main options for the minimally invasive hernia surgeon. Regarding IPOM, Allison et al. [11] studied robotic repair with intracorporeal fascial closure. Their study included 13 patients with M1 and M2 ventral hernias; the surgery consisted of the closure of the defect using a running O-absorbable suture. The average operating time was 131 min, while the mean hospital stay was 2.4 days, and only one patient experienced a recurrent hernia. On the other hand, Warren et al. [14] described robotic retromuscular and transabdominal rectus (r-TAR) release repair, comparing it with the conventional laparoscopic repair. In this technique, the surgeons incised the posterior fascia up to the semilunar line, followed by eventual rectus muscle release. In the reconstructive period, the mesh was placed in the retrorectus and preperitoneal space; at least 5 cm of overlap was the goal.

In this single-institution study, 103 laparoscopic ventral hernia operations were compared to 53 robotic retromuscular ventral hernia procedures. Several outcomes were examined. For example, the operative times were considerably longer with the robotic approach, with 245 min, as compared to 122 min for laparoscopic procedures. Concerning short-term outcomes such as seroma, it occurred at a higher rate in the robotic-treated cohort.

Liang et al. [15] published a systemic review of the best practices for ventral hernia repair, collecting robotic and laparoscopic experiences so as to determine a consensus. Fascial closure and minimally invasive techniques have evidence-based benefits, and robotics help to facilitate this. Some debates were noted regarding the excision of the sac or regarding the mesh position. With regard to the position of the mesh, some studies showed a sublay position to be the best; ultimately, this decision is up to the surgeon. Indeed, if the surgeon feels that a sublay position would be better for a particular patient, a robotic retrorectus repair could be an option.

In spite of these new procedures, open ventral hernia repair (OVHR) remains a valid and safe option due to patient comorbidities, previous abdominal surgeries, and contraindications for laparoscopic and robotic ventral hernia repair. In OVHR, different techniques are used, such as anterior component separation (ACS), posterior component separation (PCS), or transversus abdominal muscle release (TAR) [16,17,18]. Despite the new and evolving techniques, OVHR was chosen as the first surgical treatment in the following described cases.

The aim of the current study was to retrospectively analyze primary outcomes such as chronic pain and hernia recurrence at 48 months (long-term follow-up) after surgery via OVHR. In this way, we observed how specific factors and patient profiles could determine the failure of surgery and the duration of chronic pain.

## 2. Materials and Methods

Between January 2017 and December 2019, one hundred and eleven (111) consecutive patients with different and complex ventral hernias were treated via OVHR at a single tertiary hospital center.

ECG, chest X-rays, blood sample tests, and preoperative characterization were performed; moreover, pulmonary function tests (PFTs) and abdominal wall CT scans were performed.

The abdominopelvic cavity volume (ACV) and hernia sac volume (HSV) were calculated to accurately perform tailored surgery. After this, every midline incisional hernia (M1–M5) with a correlative size (W1–W3) was detected (Table 1). Inclusion (age between 18 and 90 years old, incisional ventral hernias, and OVHR with mesh) and exclusion criteria (emergency surgery, immunodeficient/oncologic patients, pregnant/breastfeeding women) were used (Table 2).

Informed consent was obtained from all patients according to the 1964 Helsinki Declaration; furthermore, for this observational, retrospective study, the “Pugliese-Ciaccio” Research Ethics Committee confirmed that no ethical approval was needed.

The primary outcomes (recurrence rate and postoperative chronic pain) and secondary outcomes (sensation and rejection phenomena and movement limitations) were evaluated by scheduled controls. Regarding primary outcomes, the follow-up was performed by clinical controls via physical examinations at 48 months after surgery, examining chronic pain and the recurrence rate. In cases of hernia recurrence, ultrasound or CT evaluation was performed. In the same clinical controls, the secondary outcomes (sensation and rejection phenomena) were studied, verifying the sensation of the foreign body on the flank or at the midline incision site.

### 2.1. Patient Data

A total of 111 patients (59 males and 52 females) were included in the current study. The median age was 59 years (18–86), and the average BMI was 32.15 for women and 33 for men (Table 3). The main comorbidities were COPD (15%) and type II diabetes mellitus (24%) (Table 4).

According to the EHS classification [3], the most frequent type of hernia was the M2 type (32%), followed by the M3 type (23%). The width (W) parameter was represented by W1 (22%), W2 (52%), and W3 (26%).

### 2.2. Surgical Technique

Below, the surgical technique regarding the most represented hernias (M2 and M3 type) is described. Each patient lay in the supine position, and, after general anesthesia, a midline incision was created. The Rives–Stoppa technique was chosen as the surgical procedure, combined with the adoption of tension-free, suture-less surgical methods for groin hernias, as described by Trabucco [19].

First, the rectus anterior fascia was set free from the subcutaneous layer across its entire length, revealing a “white plane”. Therefore, bilateral longitudinal incisions were created on the anterior fascia to render the rectal muscles visible; then, the rectus muscles were separated from the posterior fascia. With respect to the dissection of the rectus muscles from the posterior fascia, it was mandatory to perform gentle maneuvers, paying attention to the perforator vessels originating from the superior and inferior epigastric vessels. The hernial sac was replaced in the abdominal cavity; then, the posterior fascia was reconstructed, realizing a “new floor” through an overlapped double suture with non-adsorbed Prolene 0.

Indeed, the rigid monofilament polypropylene mesh (223 g/m^2^) was located free from any stitches upon the “new floor”, avoiding tension and allowing its fixation due to abdominal pressure, which was distributed equally across the entire surface according to Pascal’s law [20]. For anterior fascia repair, an overlapped double Prolene 0 closure was performed; the ACS method by Ramirez was used to move the rectus anterior sheaths closer to the anterior sheath in ten incisional hernias (2 type M4, 3 type M3, and 5 type M2).

In this way, intra-abdominal pressure (IAP), combined with the fibrosis process, derived from the interaction between the polypropylene mesh and tissues, fixed the mesh and rebuilt the abdominal wall anatomy. The dimensions of the polypropylene mesh were based on the defect size; in particular, for the W3 and M2 + M3 types, a mesh of 11 × 14 cm was applied. In only five patients, the mesh was fixed to the ensiform apophysis, after dissecting the “fatty rhomboid”, due to the presence of Swiss cheese epigastric I.H.

## 3. Results

### Postoperative Controls

Routinely, each patient underwent a specific protocol of serial control, which was subdivided into three serial steps as described. For short-term follow-up, this was performed five days, fifteen days, and ninety days after surgery, while periods of 6, 12, and 48 months were used for long-term follow-up. In this study, we retrospectively evaluated the primary and secondary outcomes of OVHR only at 48 months after surgery in 111 patients, due to the lack of long-term follow-up data in the literature.

The primary endpoint evaluation was based on the visual analog scale (VAS) score to estimate the pain intensity from zero (pain absence) to 10 (pain peak) at specific trigger points (midline, flanks, and hypogastrium). Another primary endpoint was the recurrence rate (presence [YES]/absence [NO]), which was determined by performing physical examinations or ultrasound exams/CT scans.

Regarding secondary endpoints, we analyzed sensation and rejection phenomena and movement limitations during intensive physical activity using a Likert scale (0–5 points), parameterized as follows.
12345NeverRarelySometimesOftenAlwaysCompletely agreeAgreeNeutralDisagreeCompletely disagree

**Figure 1:**
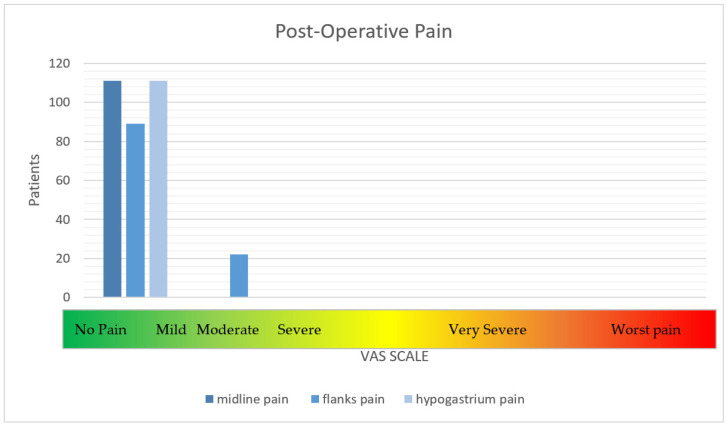
The intensity of post-operative pain.

First, a clinical examination was performed, and 10% of the patients were reached by telephone. The intensity of pain at specific trigger points, namely the flanks, midline, and hypogastrium, was assessed. The VAS was identified as the best tool for the classification of patients. At the long-term follow up, only 20% of patients reported moderate pain (4–6) along their flanks (Figure 1); on the other hand, recurrence was observed in 5% of patients, as determined by CT of the abdominal wall (Table 5).

Concerning secondary outcomes, no patient described sensation or rejection phenomena; otherwise, 18% referred to movement limitations during sports and 7% during long walking (Table 6).

## 4. Discussion

I.H. represents a surgical challenge, even for expert surgeons, for different reasons. The first reason is the choice of the mesh type due to the patient profile (comorbidities, BMI, and hernia width); the second reason is the rebuilding of the abdominal wall in complex reconstructions. In the past, simple sutures were performed to repair ventral hernias, but this was associated with a high recurrence rate; subsequently, the introduction of meshes largely reduced hernia recurrence. Despite the fact that there are several improved techniques to repair ventral hernias, in this study, a particular OVHR technique was defined using the Rives–Stoppa technique combined with Trabucco’s principles to better fix the mesh.

This study highlights the importance of Trabucco’s principles applied to ventral hernia repair in Rives–Stoppa reconstruction. Moreover, our experience demonstrates the efficacy of a semi-rigid mesh in recurrence prevention.

Indeed, in this study, a macroporous (223 g/m^2^), non-absorbable, semi-rigid, monofilament polypropylene mesh was used for the following reasons. First, the semi-rigid component prevents the mesh from moving to uncorrected positions, avoiding stitch apposition and the consequent pain at trigger points. Regarding the polypropylene aspect, Usher et al. described a low recurrence rate in hernias repaired through polypropylene meshes [4]. Following this, several studies have stressed the advantages of polypropylene meshes, including native tissue ingrowth and the speed of the fibrosis process [21,22]. The non-absorbable and macroporous parts facilitate the fibrosis process and improve the prognosis of the patient.

Regarding the appropriate surgical side, the tension-free suture-less technique, introduced by Trabucco, takes advantage of intra-abdominal pressure (IAP) to fix the mesh to the abdominal wall, applying equal pressure across the entire new surface. As reported, the semi-rigid polypropylene component and the tension-free suture-less principles, combined with IAP, can be used to fix the mesh in different types of patients and hernias.

The IAP varies between 5 and 7 mmHg, rising both in the supine and prone positions in a healthy person, whereas, in obese patients, the IAP can increase to between 7 and 14 mmHg [23,24]. Although the IAP is greater in obese patients, and elevated obesity remains a widely known risk factor for hernia recurrence, the mesh did not adhere to the abdominal wall in these patients due to microvascular defects.

Despite the inclusion of obese patients, in this study, it was demonstrated that IAP is the best natural fixing tool as opposed to stitches, glue, or spiral tracks.

Regarding the choice of the surgical technique, there is still large debate about which type of surgery is more suitable: robotic, laparoscopic, or traditional. Even if robotic surgery is accompanied by less chronic postoperative pain and it is widely accepted, there are some controversies, such as higher costs, a larger learning curve, and longer operative times [25,26].

Currently, there is no strict follow-up time for the evaluation of the recurrence rate or long-term complications after surgery. For instance, Köckerling et al. demonstrated that the range of time to observe early and late recurrence was 5 years; meanwhile, some authors, such as Rios A. et al., have noted that the ideal time could be 10 years [27,28]. Despite these points of view, in our experience, 4 years of follow-up demonstrated both late complications and recurrence, demonstrating that this time period satisfied the main goals.

The follow-up scheme was based on clinical controls and telephone interviews (only in 10% of patients). After the follow-up, the primary endpoints were the following: the recurrence rate and the incidence of postoperative chronic pain. Concerning the recurrence rate, we observed a low rate (5%), indicating that it was observed mainly in patients with diabetes mellitus type II (DMTII) and an elevated BMI [29,30,31].

The aspect of postoperative chronic pain was the most interesting. Indeed, after 48 months, 20% of patients with large hernias (W3) experienced mild pain along the flanks, probably due to mesh rubbing during abdominal contraction during heavy activities (sports, intensive labor). In fact, mild pain was observed 25% of patients, who experienced movement limitations during sports (18%) and long walking (7%), likely because of mesh rubbing.

Despite the encouraging results obtained with laparoscopic and robotic techniques, this study shows that the open approach remains a safe option to manage complex and less complex cases of I.H. in terms of the recurrence rate and chronic pain. In particular, chronic pain was experienced by a small number of patients compared with the total, demonstrating the efficacy of the open technique in reducing this long-term complication. Nevertheless, this work analyzed only open procedures performed by the same surgeons (F.A. and G.A.), representing a limitation in the assessment of the long-term complications. On one hand, the reproducibility of the technique by the same surgeons allowed shorter surgical times, greater technical confidence during the surgery, and fewer complications; on the other hand, in future case–control studies, different surgical choices could be offered to patients.

## 5. Conclusions

This was a retrospective, observational study of 111 patients who underwent OVHR with a standardized technique (the Rives technique with Trabucco principles) performed by the same surgeons (F.A. and G.A.). The technique described above is well established and especially suitable for complex incisional hernias, enabling the restoration of the abdominal wall anatomy without notable early and late complications. Surgical treatments require a standardized technique to reduce the operative time and postoperative comorbidities; on the other hand, in this study, each patient received the best treatment according to a tailored surgery. Moreover, this study included an adequate statistical analysis, using binary variables such as the recurrence rate and a pain scale (VAS scale and Likert scale) regarding chronic pain measurement. In future case–control studies, including laparoscopic procedures, it could be useful to perform a more comprehensive statistical investigation.

## Figures and Tables

**Table 1:** Incisional hernia classification.

	Type		*n*
Midline	subxiphiodal	M1	20
	epigastric	M2	35
	umbilical	M3	25
	infraumbilical	M4	10
	suprapubic	M5	10
	epigastric + umbilical	M2 + M3	11
	**Size**		***n***
Width	<4 cm	W1	24
	≥4–10 cm	W2	58
	≥10 cm	W3	29

**Table 2:** Enrolment criteria.

Inclusion Criteria	Exclusion Criteria
Age between 18 and 90	Emergency surgery
Incisional ventral hernias (M1–M5)	Immunodeficient or oncologic patients
Open ventral hernia repair with mesh	Pregnant women/breastfeeding women

**Table 3:** Demographic characteristics of the patient population.

	*n*
Age (mean)	62 (18–86)
Male	59 (53.15%)
Female	52 (46.84%)
Body mass index, male (mean)	33 (25–39)
Body mass index, female (mean)	32.15 (24–37)
Recurrent incisional hernia	6 (5%)

**Table 4:** Main patient comorbidities.

Type	*n*
Pulmonary disease (COPD, asthma)	17 (15%)
Diabetes mellitus type II (adult)	27 (24%)

**Table 5:** Postoperative complications (at 48 months).

Variable	*n*
Postoperative VAS, midline	0
Postoperative VAS, flanks	22 (20%)–VAS 4–6
Postoperative VAS, hypogastrium	0%
Recurrence	6 (5%)

**Table 6:** Secondary endpoints (at 48 months).

Variable	*n*
Sensation phenomena	0 (0%)
Rejection phenomena	0 (0%)
Movement limitations during sports	20 (18%)
Movement limitations during long walking	7 (7%)

## Data Availability

The raw data supporting the conclusions of this article will be made available by the authors on request.
